# Nutrition and Growth

**DOI:** 10.4274/jcrpe.v1i4.39

**Published:** 2010-12-08

**Authors:** Fima Lifshitz

**Affiliations:** 1 President, Pediatric Sunshine Academics, Inc; Director of Pediatrics & Senior Nutrition Scientist, Sansum Medical Research Institute, Santa Barbara, CA, USA and Professor of Pediatrics, Emeritus, Downstate Medical Center, State University of New York, Brooklyn, NY, USA; Former Professor of Pediatrics, Cornell University Medical College, New York, NY & University of Miami, Miami, FL, USA; +805-687 8038+805-682 3332DrLfshtz@aol.comPediatric Sunshine Academics, Inc. Director of Pediatrics & Senior Nutrition Scientist Sansum Medical Research Institute Santa Barbara, CA, USA

**Keywords:** nutrient intake, short stature, Nutritional growth retardation (NGR), growth retardation, metabolic rates, erythrocyte Na+, K+- ATPase activity

## Abstract

Nutrition plays a fundamental role in determining the growth of individuals. An appropriate growth progression is considered a harbinger of adequate nutrient intake and good health. On the other hand growth deceleration with or without short stature may indicate inadequate nutrition, even when there is no body weight deficit for height. Nutritional growth retardation (NGR) is most prevalent in populations at risk of poverty. However in affluent communities patients with NGR are often referred to the specialist because of short stature and delayed sexual development. The diagnosis may be overlooked and/or be established after exhaustive evaluations, if the pattern of weight progression over time is not considered. Patients with so-called idiopathic short stature may present diminished nutrient intake and decreased IGF-I levels, however their nutritional status and body weight progression patterns are usually not addressed by pediatric endocrinologists. NGR patients may cease to gain appropriate weight and fail to grow in height, even without exhibiting body weight deficits for height. They adapt to decreased nutrient intake by decreasing growth progression and thereby achieve equilibrium by decreasing the nutrient demands. This occurs by diminishing their metabolic rates and erythrocyte Na+, K+- ATPase activity, however they may not present alterations in other clinical biochemical markers of malnutrition. Therefore accurate weights and heights plotted on the growth chart over time are necessary to detect NGR. Nutritional rehabilitation is accompanied with catch up growth, though it may be difficult to change the dietary habits of adolescents who exhibit NGR.

**Conflict of interest:**None declared.

Growth is the fundamental physiologic process that characterizes childhood. It should be closely monitored by pediatricians and families alike as a benchmark of a child’s health. Similarly, secular trends in growth patterns are followed as indicators of children’s health on a population level. Growth can be worrisome along two variables: height (short stature) and velocity (growth failure).^1^ Height involves a measurement of linear stature at a single point in time and compares it to expected norms. The norms are usually provided by the general population as depicted in growth charts ([link:www.cdc.gov/growthcharts]www.cdc.gov/growthcharts[/link]).^2^ Short stature and growth failure frequently, but not always, occur together. For example, a healthy child of short parents will have short stature but not growth failure; he or she will grow at normal velocity towards a lower genetic potential. Conversely, a child of very tall parents can have growth failure, but still be taller than the cut-off for short stature of the general population.

Multiple diseases can present solely with growth failure, not necessarily with short stature. Included are non-endocrine diseases i.e. as celiac disease, cystic fibrosis, renal disease and HIV infection.^1^ These alterations share a common pathophysiological process in regards to growth failure, namely malnutrition. Non organic causes leading to decreased food intake may also result in poor growth and short stature. Failure to asses a patients’ nutritional intake can lead to unnecessarily delayed or missed NGR diagnoses. The clinical outcome of many nutritional alterations depends on the timeliness of diagnosis and treatment.^3, 4^

The single most important cause of growth retardation worldwide is povertyrelated malnutrition. When suboptimal nutrition is continued for prolonged periods of time, growth stunting occurs as the main clinical phenotype.^3, 4^ However nutritional growth retardation (NGR) is a frequently under-appreciated entity in pediatric endocrine practices in the United States. Poverty-related malnutrition is less common than in developing nations, and if anything, the current major health crisis is the obesity epidemic. Partly in response to the obesity around them, a subset of American youths, many from suburban upper middle class, restrict their nutrient intake and develop NGR and delayed sexual development.^1^ This decreased intake is on the continuum of weight gain problems; it is insufficient to support normal growth but it does not include a distorted body image as occurs in eating disorders.^5^

Children with NGR are generally referred to the pediatric endocrinologist because of short stature or delayed puberty. Therefore, pediatricians and pediatric endocrinologists need to recognize NGR and become familiar with its causes and treatment. Although the importance of evaluating the pattern of stature increments throughout life in the differential diagnosis of short stature cannot be over-emphasized, carefully assessing the progression of body weight is equally relevant to be able to recognize NGR. Longitudinal assessment of both height and weight is required.^6-9^

An increasing number of children on stimulant medications are being referred to the pediatric endocrinologist for short stature evaluation. Stimulant medication for the treatment of attention deficit hyperactivity disorder (ADHD) has long been suspected of adversely affecting linear growth, since it is well known that these medications produce anorexia and poor nutrient intake. A cooperative growth paper reviewed 29 cohort studies of children treated with methylphenidate or dexamphetamine.^10^ The most sensitive studies measured growth progression before and after the period of treatment, and eight of these 16 studies showed an attenuation of growth on stimulants. In the most rigorous study, 540 children, 7-9 yr old, with ADHD were randomly assigned to different treatment groups for up to 24 months. The behavioral effectiveness of medication use was greatest among children who ingested medications throughout the 24-month observation period. Those who stopped taking their medication and those who did not ingest them consistently showed increasing behavioral problems. However, there was significant growth deterioration among children who took the medication for the longest periods. After 2 years’ treatment, height was suppressed by a mean of –1.94 cm and deficits in weight gain were even larger. The authors concluded that consistent treatment with stimulant medication was associated with maintenance of behavioral effectiveness but continued growth suppression.^11^ The somewhat larger deterioration observed in body weight may be due to the anorexic effects of these medications. Suboptimal nutrition appears to be an underlying cause of stimulant-mediated growth faltering.

The classic anthropometric criteria for NGR stipulate low weight for age with minimal deficits in weight for height. By these cross-sectional criteria, it may be difficult to differentiate NGR children from those with familial short stature or constitutional growth delay.^6-9^ Only the longitudinal progression of body weight and height can more clearly reveal NGR, which may occur even when there is weight-for-height excess.^12^ The distinguishing feature is a delay in linear growth and puberty resulting from inadequate weight gain. Thus, although concern is intensified when weight or height measurements fall below the 5^th^ percentile, growth failure expressed as deterioration across percentiles of weight and height may also indicate NGR even when the child is still above the 5^th^ percentile. With nutritional rehabilitation, catch-up growth is usually achieved.^1, 7-9^

Analysis of body weight progression may be the most important clue for diagnosing NGR in patients with short stature. Calculation of theoretical weights and heights based on previous growth percentiles may be used to quantitatively compare current anthropometric indices with previously established patterns of weight and height progression.^1, 7, 8, 9^ Theoretical weight is defined as the weight the patient should have had at the time of the examination, if the patient had continued to gain weight along the percentile previously established during the pre-morbid growth period. Body weight-for-height deficits are not common in NGR, but the body weight is often deficient relative to the theoretical weight. In contrast, short patients without NGR, such as those with constitutional growth delay, continue to gain weight along established percentiles and the body weight at the time of assessment is equal to the theoretical body weight. A fall in growth associated with a poor rate of weight gain indicates NGR, even without an appreciable weight-for-height deficit.^1, 9, 12^

Patients with NGR do not appear wasted, and the usual biochemical parameters of nutritional status, including serum levels of retinol-binding protein, pre-albumin, albumin, transferrin, and triiodothyronine (T3) levels, do not differentiate NGR patients from those with familial or constitutional short stature. Other indices of malnutrition, such as the urinary creatine-height index or urinary nitrogen/creatinine ratio, do not usually demonstrate abnormalities. The reason is that NGR patients have adapted to their suboptimal nutritional intake and they maintain homeostasis by decreasing growth, thereby reaching equilibrium with preservation of biochemical nutritional markers.^13^

Although fasting and protein-calorie malnutrition have been shown to lower circulating IGF-I levels in humans and rodents, IGF-I levels may not differentiate NGR patients from those with familial and/or constitutional short stature. The degree of nutritional insufficiency in NGR is not as severe as that observed in protein-calorie malnutrition or fasting, and may impair growth by altering other cellular mechanisms without affecting the serum IGF-I levels as discussed below. Because the energy restriction is mild, and NGR children consume sufficient dietary protein, IGF-I concentrations may be preserved within a range appropriate for bone age development. Likewise, rats consuming diets containing 15% protein and 90% of the total energy requirements, maintained the IGF-I concentrations within normal ranges.^14^

We reported that NGR patients show decreased activity of erythrocyte Na^+^,K^+^- ATPase compared with familial and/or constitutional short stature patients.^13^ This enzyme is involved in the active transport of sugars and amino acids and in cellular thermogenesis, normally accounting for approximately one-third of the basal energy requirements. Reduced energy intake lowers the basal metabolic rate and decreases Na^+^, K^+^-ATPase activity. Because anthropometric parameters may be lacking or inaccurate and usual biochemical markers may not be sufficient to detect NGR, a more sensitive test is required for the diagnosis of NGR. Erythrocyte Na^+^, K^+^-ATPase activity may offer such a diagnostic tool. However, to date, this assay has not been widely available for clinical purposes, is cumbersome, and has only been applied on a research basis.^13, 14^

Growth deceleration is the adaptive response to suboptimal nutrition, so patients with NGR have achieved equilibrium between their genetic growth potential and their nutritional intake. Diminished growth brings the nutrient demands into balance with the nutritional intake, without adversely affecting biochemical or functional homeostatic measures. Of course, there are limits to these adaptive possibilities. If nutritional deprivation becomes more severe, or when acute malnutrition is superimposed on the chronic suboptimal state, there will be altered anthropometric measurements, such as weight and skin fold thickness, and biochemical indices reflecting malnutrition.

The biochemical and hormonal changes associated with chronic suboptimal nutrition have been studied utilizing a rodent model. Sodium-potassium ATPase activity was reduced in rats fed less than 80% of ad-libitum energy intake.^14^ Body weight gain was preserved in sub optimally fed rats treated with recombinant human GH.^15, 16^ Furthermore, simultaneous restriction of both energy and zinc did not augment the growth deterioration of chronic suboptimal nutrition.^17^ Substitution of fat for carbohydrates led to greater body weight gains, through reduced energy expenditure and possibly decreased leptin secretion.^18^ Other changes due to chronic suboptimal nutrition included a reduction of liver weight with an increase in percent total polyunsaturates, n- 6 polyunsaturates and total unsaturates in mitochondrial lipids. Suboptimal nutrition also reduced mandibular and femur bone growth. Finally, rats sub optimally fed for three weeks showed decreased T-cell numbers in the thymus that may alter the immune system. All these studies suggest that minor biochemical and physiological changes occur during chronic suboptimal nutrition.

We have shown that diminished energy intake resulting in NGR reduces metabolic rate even before there is a loss of body weight.^19^ The rate of protein synthesis may decrease in response to a reduction in energy intake, because this process is energy expensive and accounts for 10–15% of the basal metabolic rate.^20^ It has long been known that protein catabolism is also sensitive to energy deprivation, such that reduced dietary energy sources may lead to increased nitrogen fiuxes in which protein breakdown is accelerated to provide energy.^21, 22^ Nitrogen retention markedly increases during nutritional rehabilitation;^23^ nutritional recovery also normalizes the excretion of amino acids and increases the rate of protein synthesis.^24^ In NGR, the result of the altered rates of protein turnover and nitrogen retention may be the cessation of normal growth, as an adaptive response to the decreased intake. In addition to suboptimal energy intake, various mineral and vitamin deficiencies have been implicated in the etiology of NGR.^1, 25^

More recently we studied the changes in the 24-hr metabolic and physical activity profile of rodents undergoing chronic suboptimal nutrition to assess if the metabolic adaptations contribute to the preservation of body weight gain and growth.^26^ We utilized the rodent EMTAC to conduct accurate measurements of continuous energy expenditure and physical activity in rats restricted to 80, 70 or 60% of ad-libitum energy consumed by controls. Rats that were restricted to only 80% of their ad-libitum energy intake grew at lower rates than those of the ad-libitum fed controls. Furthermore, they preserved fat-free mass, but had reduced energy expenditure and physical activity, along with increased respiratory quotients, during the dark period at night. Thus, these rats utilized their body fat and reduced their physical activity to conserve energy to preserve lean body mass and to allow some growth. However, rats fed only 70% of ad-libitum energy intake had reductions in both growth and lean body mass and had a greater magnitude in the reduction of energy expenditure and physical activity. Rats subjected to even greater amounts of energy restriction, such as those fed 60% of ad-libitum energy intake, had even greater detrimental effects such as reduced growth, loss of lean body and fat mass along with further decreases in energy expenditure and physical activity. Despite consuming only 60% of their ad-libitum energy intake, these rats still preserved 26% of their body weight gain as compared to ad-libitum fed controls. This suggests that over the course of the experiment, some essential needs of metabolism were still being met and a minimal amount of energy was still available for growth.^26^

Other physiological adaptations may contribute to the maintenance of health and body weight gain during chronic suboptimal nutrition. For example, a reduction of body temperature might be another mechanism for energy conservation, since changes in energy expenditure are directly related to body temperature.^27^ It is possible that other factors might also contribute to the preservation of metabolic homeostasis, such as alterations in erythrocyte sodium-potassium- ATPase activity.^14^ Nonetheless, it remains controversial whether decreased body size is an advantageous adaptation to a limited food supply or whether adverse health and functional impairments result. Mild energy restriction in rodents has been repeatedly shown to extend life span.^28^

However it is difficult to conceive an appropriate homeostasis that will allow optimal health and prolongation of life with levels of energy restriction that are associated with poor growth and degradation of lean body mass. Physical activity is decreased with a 20% decrease in energy consumption, and as mentioned above, energy expenditures in rats promptly decrease with a relatively mild energy restriction.^14, 18, 26^ Moreover there are other functional impairments that are more difficult to assess, such as mental capacity and learning ability that may also be compromised. Decreased growth velocitynevertheless constitutes a functional compromise per se, which should be detected and treated as early as possible.^1, 29, 30, 31^

Nutritional rehabilitation for NGR of nonorganic origin requires providing the patient with adequate caloric and nutrient intake for the restoration of previous growth patterns. Initially, estimation of energy requirements should be based on the ageand gender-specific RDA based on the patient’s theoretical weight. Adequate intake of protein usually accompanies sufficient caloric intake, but care should be taken that micronutrient intakes meet the RDA and specific deficiencies, such as iron or zinc, should be treated.^30, 31^ Some patients may not be willing or able to consume a completely balanced diet and may require a multivitamin and mineral supplement. A careful diet history can elucidate food preferences and eating patterns that need to be considered in devising an appropriate dietary plan. Our experience has been to offer general dietary suggestions rather than to prescribe a specific diet. Frequent follow-up visits provide an opportunity to revise and update dietary recommendations and to assess weight and height improvements. Although the appropriate diet can be easily determined, successful intervention requires a change in dietary behaviors and possibly health beliefs as well.^32^ Increasing the caloric density of the child’s diet often involves raising the dietary fat and providing nutrient dense foods that the patient and the family may not accept. The assurance that an appropriate nutritional intake will result in normal growth, without producing obesity, is necessary supportive therapy. This is of particular concern in the initial stages of the treatment, when weight increases rapidly before any noticeable effect on height is observed.^29, 30, 31^

The USDA guidelines for dietary intake were released, September 24 2004.^33^ The DRIs are evidence-based recommendations for planning and assessing dietary intake of apparently healthy people and the reader is encouraged to refer to the DRI book or website ([link:http://www.nal.usda.gov/fnic/etext/000105.html]http://www.nal.usda.gov/fnic/etext/000105.html[/link]) when evaluating the dietary intake of a given patient. However the USDA food guide is a simpler guideline which should serve the needs of Pediatric Endocrinologists when evaluating the quality of the dietary intake of a short child and to provide guidelines for intake to the patients. The consumption of the recommended foods from each group is a fairly good indicator of the adequacy of the dietary intake, if maintained over time.^33^

## ACKNOWLEDGEMENT

Supported by Pediatric Sunshine Academics Inc.
